# Risk factors for mortality in post-myocardial infarction patients: insights from the improve SCA bridge study

**DOI:** 10.1186/s43044-024-00505-2

**Published:** 2024-06-07

**Authors:** Dileep Kumar, Fawaz Bardooli, Wen-Jone Chen, Dejia Huang, Mullasari Ajit Sankardas, Waqar Habib Ahmed, Houng-Bang Liew, Hyeon-Cheol Gwon, Brian Van Dorn, Thomas Holmes, Amy Thompson, Shu Zhang

**Affiliations:** 1 Mohammed Bin Khalifa Specialist Cardiac Centre, Awali, Bahrain; 2 Min-Shen General Hospital, Taoyuan, Taiwan; 3 National Taiwan University Hospital, Taipei, Taiwan; 4 Madras Medical Mission, Chennai, India; 5 King Fahad Armed Forces Hospital, Jeddah, Saudi Arabia; 6 Queen Elizabeth Hospital II Clinical Research Centre, Sabah, Malaysia; 7 Samsung Medical Center, Sungkyunkwan University School of Medicine, Seoul, South Korea; 8 Medtronic Inc, Mounds View, MN USA; 9 Fu Wai Hospital Chinese Academy of Medical Sciences, Peking Union Medical College, Beijing, Mainland, China; 10 Phoenix Hospital, Abu Dhabi, United Arab Emirates

**Keywords:** Sudden cardiac death, Ischemic vs non-ischemic cardiomyopathy, Implantable cardioverter-defibrillator

## Abstract

**Background:**

Underutilization of implantable cardioverter defibrillators (ICD) to prevent sudden cardiac death (SCD) in post-myocardial infarction (MI) patients remains an issue across several geographies. A better understanding of risk factors for SCD in post-MI patients from regions with low ICD adoption rates will help identify those who will benefit from an ICD. This analysis assessed risk factors for all-cause and cardiovascular-related mortality in post-MI patients from the Improve Sudden Cardiac Arrest (SCA) Bridge Trial.

**Results:**

For the entire cohort, the overall 1-year mortality rate was 5.9% (88/1491) and 3.4% (51/1491) for all-cause and cardiovascular mortality, respectively, with 76.5% of all cardiac deaths being from SCD. A multivariate model determined increased age, reduced left ventricular ejection fraction (LVEF), increased time from myocardial infarction to hospital admission, being female, being from Southeast Asia (SEA), and having coronary artery disease to be significant risk factors for all-cause mortality. The risk factors for cardiovascular-related mortality revealed increased age, reduced LVEF, and being from SEA as significant risk factors.

**Conclusions:**

We show several characteristics as being predictors of cardiovascular-related mortality in post-MI patients from the Improve SCA Bridge study. Patients who experience an MI and present with these characteristics would benefit from a referral to an electrophysiologist for further SCD risk stratification and management and possible subsequent ICD implantation to reduce unnecessary death.

**Supplementary Information:**

The online version contains supplementary material available at 10.1186/s43044-024-00505-2.

## Background

Implantable cardioverter defibrillators remain the standard of care to prevent sudden cardiac death (SCD) in indicated patients [1]. However, the rate of ICD implants for those who need them remains low, especially in regions underrepresented in major ICD clinical trials [2, 3]. This is concerning given that SCD remains one of the most common causes of death worldwide.

Patients who experience a myocardial infarction (MI) have been shown to be at heightened risk for SCD, despite recent advancements in the management of these patients [3, 4]. Current guidelines recommend the use of ICDs in post-MI patients who have a reduced left ventricular ejection fraction (LVEF) (≤ 35%) for 40 days after MI [1, 5]. Waiting 40 days to implant post-MI is based on two major studies that showed no benefit of early ICD intervention in post-MI patients [6, 7]. However, more research is needed to better identify post-MI patients at risk of SCD to help determine who would best benefit from an ICD, especially in regions where ICD use remains low.

The Improve Sudden Cardiac Arrest (SCA) Bridge Trial aimed to identify barriers to patient referral for SCD risk stratification and management in regions with low ICD utilization [8]. Understanding risk factors for death following MI in these patients may help inform decisions on SCD risk management in regions where ICD adoption is lagging. Using data from the Improve SCA Bridge cohort, the current study aims to identify risk factors for all-cause and cardiovascular-related mortality to help identify those who may benefit from further SCD risk stratification and management.

## Methods

### Improve SCA bridge study design and eligibility

The Improve SCA Bridge Trial (ClinicalTrials.gov; Identifier: NCT03715790) was a prospectively enrolled, non-randomized, multicenter, global, post-market study aimed at identifying reasons why post-MI patients were not referred for further SCD risk stratification and management [8]. The six regions that participated in the study were: 1. Mainland China, 2. India Subcontinent (ISC, including India and Bangladesh), 3. South Korea, 4. Middle East, Africa, Central Asia and Turkey (MEACAT, including Egypt, Pakistan, Saudi Arabia, South Africa and Tunisia), 5. Southeast Asia (SEA, including Brunei, Indonesia, Malaysia, The Philippines, Singapore and Thailand), and 6. Taiwan. These regions were chosen due to their low rate of ICD therapy adoption. The full study design details have been published previously [8].

The inclusion criteria for enrollment in the study were as follows: (1) Age 18 and above (and met age requirements per local law); (2) Experienced an acute ST-segment elevation myocardial infarction (STEMI) or non‐ST-segment elevation myocardial infarction (NSTEMI) ≤ 30 days before enrollment, and [3] An LVEF < 50% measured within 14 days of the MI. Exclusion criteria are outlined in Supplemental Table 1. Follow-up visits occurred at 3, 6, and 12 months and were performed either in-person or by phone due to the ongoing COVID-19 pandemic.

**Table 1:** Baseline patient characteristics

Subject Characteristics	China N = 394)	ISC (N = 347)	Korea (N = 237)	Taiwan (N = 120)	MEACAT (N = 197)	SEA (N = 196)	Overall (N = 1491)
*Age (years)*							
Mean ± SD	63.0 ± 11.5	56.4 ± 11.2	64.5 ± 12.1	63.0 ± 11.1	57.8 ± 12.0	56.8 ± 11.3	60.2 ± 12.0
Median	63.5	56.0	64.0	62.0	57.0	57.0	60.0
Minimum–Maximum	24–97	21–90	32–90	36–92	29–90	35–93	21–97
*Gender (N, %)*							
Male	302 (76.6%)	291 (83.9%)	195 (82.3%)	99 (82.5%)	167 (84.8%)	174 (88.8%)	1228 (82.4%)
*ST elevation*							
STEMI (N, %)	257 (65.2%)	236 (68.0%)	147 (62.0%)	68 (56.7%)	132 (67.0%)	145 (74.0%)	985 (66.1%)
*NYHA classification (N, %)*							
Subject Does Not Have Heart Failure	144 (36.5%)	198 (57.1%)	205 (86.5%)	80 (66.7%)	140 (71.1%)	139 (70.9%)	906 (60.8%)
Class I	11 (2.8%)	10 (2.9%)	0 (0.0%)	1 (0.8%)	25 (12.7%)	18 (9.2%)	65 (4.4%)
Class II	76 (19.3%)	68 (19.6%)	3 (1.3%)	10 (8.3%)	14 (7.1%)	18 (9.2%)	189 (12.7%)
Class III	78 (19.8%)	14 (4.0%)	9 (3.8%)	5 (4.2%)	5 (2.5%)	10 (5.1%)	121 (8.1%)
Class IV	47 (11.9%)	5 (1.4%)	3 (1.3%)	5 (4.2%)	12 (6.1%)	8 (4.1%)	80 (5.4%)
NYHA Classification Not Available	38 (9.6%)	52 (15.0%)	17 (7.2%)	19 (15.8%)	1 (0.5%)	3 (1.5%)	130 (8.7%)
*LVEF at baseline*							
Mean ± SD	41.3 ± 6.1	39.5 ± 5.7	40.2 ± 7.8	40.6 ± 8.1	38.3 ± 5.9	37.7 ± 7.7	39.8 ± 6.8
Median	43.0	40.0	42.0	43.0	40.0	39.1	40.0
Minimum–Maximum	19–49	20–49	13–50	4–49	25–49	9–50	4–50
*Door to Balloon Time (Hours)*							
Subjects With Measure Available (N, %)	232 (58.9%)	224 (64.6%)	214 (90.3%)	96 (80.0%)	154 (78.2%)	146 (74.5%)	1066 (71.5%)
Mean ± SD	55.7 ± 112.1	29.1 ± 52.9	16.9 ± 32.9	25.3 ± 68.6	13.1 ± 30.6	58.5 ± 191.8	33.8 ± 97.0
Median	3.1	7.2	1.5	1.8	1.7	4.1	2.6
Minimum–Maximum	− 3–1234	− 12–537	− 1–231	− 79–441	− 11–191	− 11–1386	− 79–1386
*MI to Hospital Admission (Days)*							
Mean ± SD	2.3 ± 3.6	1.0 ± 1.8	0.6 ± 1.3	0.3 ± 0.6	0.4 ± 1.0	0.5 ± 0.7	1.1 ± 2.3
Median	0.8	0.3	0.1	0.1	0.1	0.2	0.2
Minimum–Maximum	-9–17	− 0–12	0–10	− 0–4	− 1–10	− 1—4	− 9–17
*Diabetes*							
Any	146 (37.1%)	153 (44.1%)	76 (32.1%)	50 (41.7%)	80 (40.6%)	59 (30.1%)	564 (37.8%)
Type I	0 (0.0%)	19 (5.5%)	5 (2.1%)	1 (0.8%)	8 (4.1%)	1 (0.5%)	34 (2.3%)
Type II	146 (37.1%)	134 (38.6%)	71 (30.0%)	49 (40.8%)	73 (37.1%)	58 (29.6%)	531 (35.6%)
Cancer	5 (1.3%)	0 (0.0%)	14 (5.9%)	10 (8.3%)	4 (2.0%)	3 (1.5%)	36 (2.4%)
COPD	8 (2.0%)	13 (3.7%)	4 (1.7%)	5 (4.2%)	1 (0.5%)	2 (1.0%)	33 (2.2%)
Renal Dysfunction	33 (8.4%)	5 (1.4%)	26 (11.0%)	17 (14.2%)	14 (7.1%)	18 (9.2%)	113 (7.6%)
CHF	0 (0.0%)	45 (13.0%)	15 (6.3%)	27 (22.5%)	3 (1.5%)	4 (2.0%)	94 (6.3%)
CAD	231 (58.6%)	169 (48.7%)	31 (13.1%)	40 (33.3%)	38 (19.3%)	30 (15.3%)	539 (36.2%)
Hypertension	207 (52.5%)	148 (42.7%)	131 (55.3%)	74 (61.7%)	87 (44.2%)	95 (48.5%)	742 (49.8%)
PVD	24 (6.1%)	0 (0.0%)	2 (0.8%)	6 (5.0%)	3 (1.5%)	0 (0.0%)	35 (2.3%)
Prior stroke	26 (6.6%)	5 (1.4%)	13 (5.5%)	9 (7.5%)	7 (3.6%)	5 (2.6%)	65 (4.4%)

*CAD* Coronary artery disease, *CHF* Congestive heart failure, *COPD* Chronic obstructive pulmonary disease, *ISC* India Subcontinent, *LVEF* Left ventricular ejection fraction, *MEACAT* Middle East, Africa, Central Asia and Turkey, *PVD* Peripheral vascular disease, *SD* Standard deviation, *SEA* Southeast Asia, *STEMI* ST-elevated myocardial infarction

### Cause of death classification

Classification for the cause of death was determined by each individual site and adjudicated by an outside clinical events committee. For the purposes of this study, deaths were classified as either SCD, non-SCD, non-cardiac death, or unknown. Regulatory reporting of deaths was completed according to local regulatory requirements.

### Study objectives

The main objective of this study was to determine risk factors for all-cause and cardiovascular-related mortality using data from the Improve SCA Bridge cohort. These risk factors could help inform decisions on SCD risk stratification and management to better determine those who would benefit from ICD therapy. Secondary objectives included all-cause and cardiovascular-related mortality in STEMI and NSTEMI patients, separately. We also reported the causes of death and Kaplan–Meier estimated 1-year all-cause and cardiovascular-related mortality rates.

### Statistical analysis

Quantitative data were reported as the mean and standard deviation while categorical data was reported as the number and percent ratio. Kaplan–Meier estimates of the survival function were used to determine the 1-year rate of all-cause mortality and SCD in our patient population. For the risk factor analysis, a total of 21 patient characteristics were used as candidates (same as baseline characteristics in Table 1). For the sake of comparison, potential risk factors were first assessed using a univariate Cox proportional-hazards model. All 21 predictors were then entered into a multivariate Cox proportional-hazards model applying a backward selection process. Predictors were removed from the multivariate model if their *p*-value was greater than 0.15. At the conclusion of the selection process, any predictors with a *p*-value < 0.05 were considered significant. The univariate and multivariate Cox analyses were performed for the entire patient cohort using all 21 characteristics, and for STEMI and NSTEMI populations separately, using 20 predictors (STEMI status was naturally excluded as a predictor). A multivariate Cox proportional-hazards model assumes that the effect of different variables on survival is constant over time. All statistical analysis was performed using SAS software, version 9.4 (SAS Institute Inc., Cary NC).

### Ethics statement

The primary Improve SCA Bridge Study and this sub-analysis of Improve SCA Bridge were conducted in compliance with the Declaration of Helsinki, and the protocol was approved by the Ethics Committee at each participating site before enrollment.

## Results

### Baseline characteristics

**Fig. 1:**
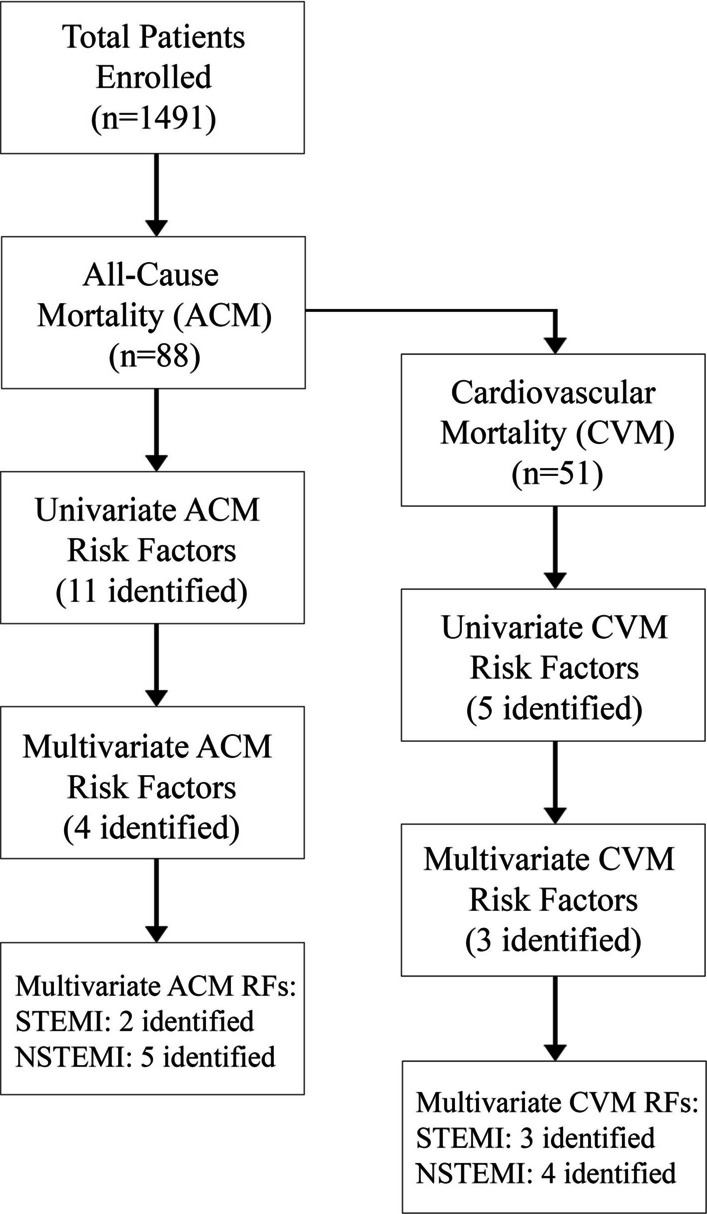
Risk factors analysis workflow and summary of results. Workflow of sample sizes included in the analysis and general results from each analysis. *ACM* All-cause mortality, *CVM* Cardiovascular mortality, *RF* Risk factors

A total of 1491 post-MI patients were enrolled in the Improve SCA Bridge study (Fig. 1). Baseline characteristics for all patients can be found in Table 1. The average age of enrolled patients was 60.2 ± 12 years, with 82.4% being male, 35.6% having type 2 diabetes, and 49.8% presenting with hypertension (Table 1). Nearly two-thirds (66.1%) of all patients had an MI that was ST-elevated (STEMI) with SEA having the highest percentage of STEMI patients among all regions at 74% (Table 1). In most geographies the time from MI to hospital admission was under 24 h, with the exception being Mainland China having a mean time of 2.1 ± 3.4 days (Table 1).

### Risk factors for all-cause mortality

**Fig. 2:**
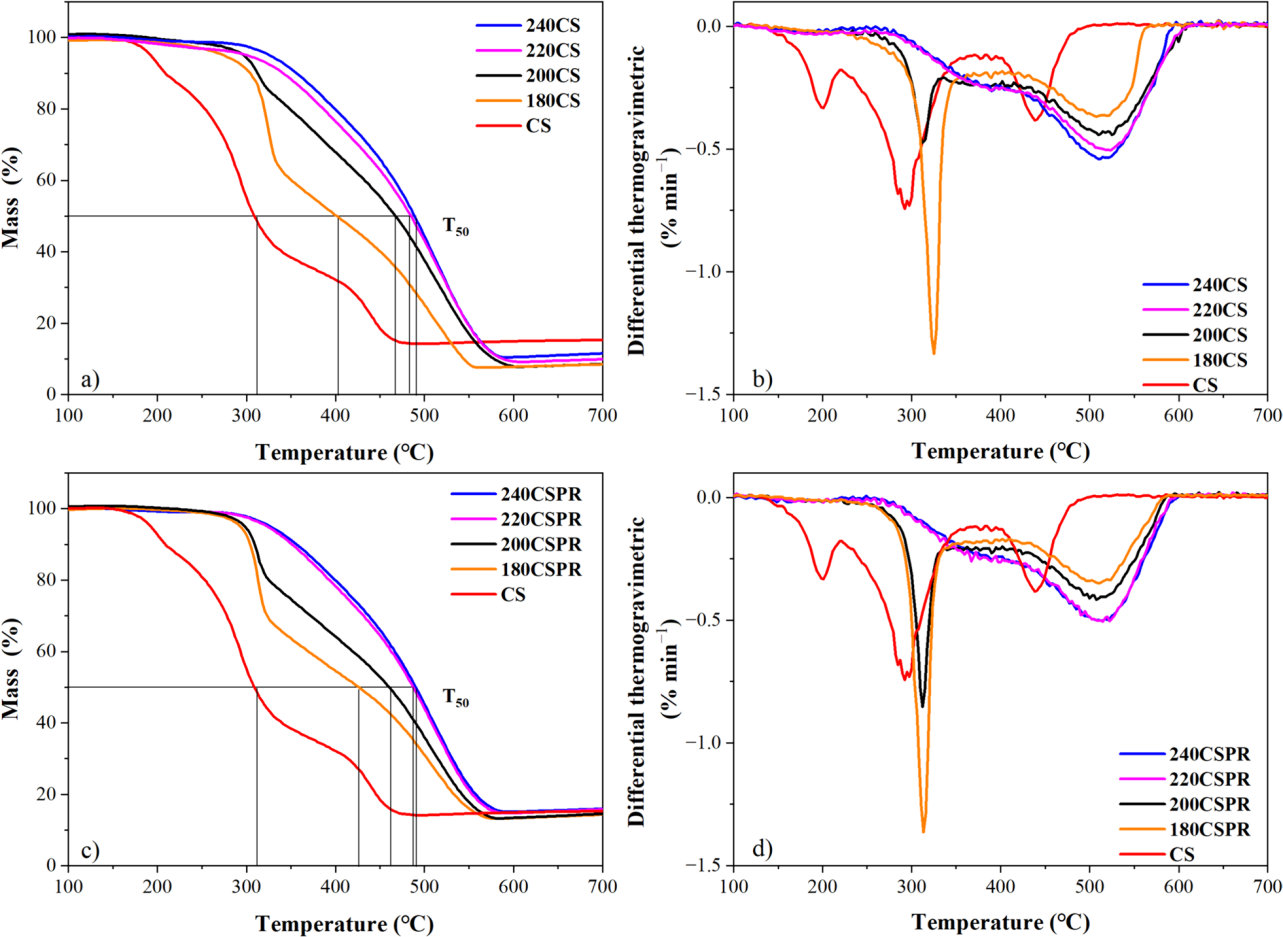
Kaplan–Meier plots for all-cause and cardiovascular mortality. Kaplan–Meier curves for all-cause mortality **A** and sudden cardiac death **B** at 1 year. Close-up graphs are given for both **A** and **B** where the y-axis begins at 80% instead of 0%

Mortality of any etiology occurred in 88 patients (5.9% of all enrolled patients) during the study period (Table 2). The Kaplan–Meier estimated all-cause mortality at 1 year was 7% (Fig. 2A). Twenty-one characteristics were screened as possible risk factors for all-cause mortality, of which 12 were significant via a univariate model (Table 3). A separate multivariate model revealed six independent risk factors for all-cause mortality which included: increased age (*p* = 0.0001), low LVEF (*p* = 0.004), increased time from MI to hospital admission (*p* < 0.013), female gender (*p* = 0.017), being from SEA (*p* = 0.006), and presence of CAD (*p* = 0.011) (Table 3).

**Table 2:** All-cause and cardiovascular mortality rates

Mortality	China (*N* = 394)	ISC (*N* = 347)	Korea (*N* = 237)	Taiwan (*N* = 120)	MEACAT (*N* = 197)	SEA (*N* = 196)	Overall (*N* = 1491)
All-cause mortality (N, %) Sudden cardiac death Non-sudden cardiac death Non-cardiac death Unknown classification	40 (10.2%) 13 (3.3%) 1 (0.3%) 9 (2.3%) 17 (4.3%)	12 (3.5%) 9 (2.6%) 2 (0.6%) 1 (0.3%) 0 (0.0%)	7 (3.0%) 4 (1.7%) 2 (0.8%) 0 (0.0%) 1 (0.4%)	0 (0.0%) 0 (0.0%) 0 (0.0%) 0 (0.0%) 0 (0.0%)	9 (4.6%) 3 (1.5%) 4 (2.0%) 1 (0.5%) 1 (0.5%)	20 (10.2%) 10 (5.1%) 3 (1.5%) 3 (1.5%) 4 (2.0%)	88 (5.9%) 39 (2.6%) 12 (0.8%) 14 (0.9%) 23 (1.5%)
Cardiovascular mortality (N, %)	14 (3.6%)	11 (3.2%)	6 (2.5%)	0 (0.0%)	7 (3.6%)	13 (6.6%)	51 (3.4%)

*ISC* India Subcontinent, *MEACAT* Middle East, Africa, Central Asia and Turkey, *SEA* Southeast Asia

**Table 3:** Risk factors for all-cause mortality in all patients

			Univariate model		Multivariate model*	
Characteristics	*N* (Counts)	Mean value (N = 1491)	Hazard ratio (95% CI)	*P*-Value	Hazard ratio (95% CI)	*P*-Value
Age (years)		60.2	1.046 (1.028, 1.065)	< 0.0001	1.037 (1.018, 1.057)	0.0001
LVEF (%)		39.8	0.951 (0.926, 0.976)	0.0001	0.961 (0.935, 0.987)	0.0039
Door to Balloon Time (Hours)		33.8	1.000 (0.997, 1.003)	0.8724		
Time MI to Hospital Admission (Min)		1572.3	1.000 (1.000, 1.000)	0.0002	1.000 (1.000, 1.000)	0.0125
STEMI	984		0.508 (0.334, 0.772)	0.0015	0.724 (0.468, 1.120)	0.1465
Female	263		2.441 (1.565, 3.807)	< .0001	1.774 (1.110, 2.834)	0.0165
China	394		2.625 (1.724, 3.998)	< .0001		
Korea	237		0.469 (0.216, 1.014)	0.0543		
SEA	196		1.942 (1.180, 3.198)	0.0091	2.547 (1.489, 4.355)	0.0006
ISC	347		0.468 (0.254, 0.860)	0.0145		
MEACAT	197		0.685 (0.344, 1.366)	0.2828		
Taiwan	118		No Patients Died	0.9743		
Diabetes	564		1.668 (1.098, 2.533)	0.0165	1.420 (0.924, 2.182)	0.1097
Cancer	36		0.473 (0.066, 3.395)	0.4565		
COPD	33		1.588 (0.502, 5.023)	0.431		
Renal	113		2.662 (1.527, 4.641)	0.0006		
CHF	94		0.325 (0.080, 1.320)	0.116	0.258 (0.063, 1.063)	0.0607
CAD	539		2.000 (1.316, 3.040)	0.0012	1.771 (1.143, 2.743)	0.0105
Hypertension	742		1.439 (0.942, 2.197)	0.0919		
PVD	35		0.984 (0.242, 3.997)	0.9817		
Prior stroke	65		1.948 (0.900, 4.215)	0.0906		

*CAD* Coronary artery disease, *CHF* Congestive heart failure, *CI* Confidence interval, *COPD* Chronic obstructive pulmonary disease, *ISC* India Subcontinent, *LVEF* Left ventricular ejection fraction, *MEACAT* Middle East, Africa, Central Asia and Turkey, *PVD* Peripheral vascular disease, *SEA* Southeast Asia, *STEMI* ST-elevated myocardial infarction

*In multivariate analysis, only 2 factor were recognized for all cause mortality. However, in univariate analysis 6 factors were idntified

### Risk factors for cardiovascular-related mortality

Cardiovascular mortality represented 57.9% (51/88) of all deaths during the study, 76.5% (39/51) of which were a result of SCD (Table 2). The Kaplan–Meier estimate of 1-year SCD was 3.9% (Fig. 2B). Of the 21 potential risk factors analyzed for cardiovascular mortality, three were significant at alpha = 0.05 after applying the multivariate model including increased age (*p* = 0.023), low LVEF (*p* = 0.0002), and being from SEA (*p* = 0.009) (Table 4).

**Table 4:** Risk factors for cardiovascular mortality in all patients

			Univariate model		Multivariate model*	
Characteristics	*N* (Counts)	Mean Value (*N* = 1491)	Hazard Ratio (95% CI)	*P*-Value	Hazard Ratio (95% CI)	*P*-Value
Age (years)		60.2	1.036 (1.012, 1.060)	0.0027	1.028 (1.004, 1.053)	0.0232
LVEF (%)		39.8	0.930 (0.901, 0.959)	< 0.0001	0.939 (0.909, 0.970)	0.0002
Door to balloon time (Hours)		33.8	1.000 (0.996, 1.004)	0.9607		
Time MI to hospital admission (Min)		1572.3	1.000 (1.000, 1.000)	0.1514		
STEMI	984		0.648 (0.372, 1.127)	0.1245		
Female	263		2.036 (1.114, 3.718)	0.0208	1.758 (0.928, 3.328)	0.0833
China	394		1.149 (0.621, 2.127)	0.6589	(Reference for Geographic Regions)	
Korea	237		0.718 (0.306, 1.683)	0.4457		
SEA	196		2.246 (1.196, 4.216)	0.0118	2.395 (1.241, 4.621)	0.0092
ISC	347		0.845 (0.433, 1.649)	0.622		
MEACAT	197		0.977 (0.440, 2.171)	0.9553		
Taiwan	120		No Patients Died			
Diabetes	564		1.481 (0.854, 2.566)	0.1619		
Cancer	36		No Patients Died			
COPD	33		1.827 (0.444, 7.514)	0.4033		
Renal	113		2.367 (1.113, 5.034)	0.0253		
CHF	94		0.579 (0.141, 2.381)	0.4486		
CAD	539		1.684 (0.973, 2.917)	0.0627	1.679 (0.956, 2.946)	0.0711
Hypertension	742		1.265 (0.728, 2.195)	0.4043		
PVD	35		1.716 (0.417, 7.055)	0.4542		
Prior stroke	65		0.913 (0.222, 3.756)	0.9000		

Abbreviations same as in Table 3

### Mortality risk factors in STEMI vs. NSTEMI patients

Baseline characteristics separated by STEMI and non-STEMI patients can be found in Supplemental Table 2. In STEMI patients, six factors were identified as significant for all-cause mortality with the univariate model, while only two factors, low LVEF (*p* < 0.0001) and presence of CAD (*p* = 0.002), were significant at alpha = 0.05 after the multivariate analysis (Supplemental Table 3). As with all-cause mortality, low LVEF (*p* < 0.0001) and presence of CAD (*p* = 0.008) were risk factors for cardiovascular-related mortality in STEMI patients, in addition to the presence of renal disease (*p* = 0.037) (Supplemental Table 4).

Univariate analysis revealed seven risk factors for all-cause mortality in NSTEMI subjects. Of these, four were significant after multivariate analysis: increased age (*p* < 0.0001), increased time from MI to hospital admission (*p* = 0.003), being from SEA (p = 0.0002), and having diabetes (*p* = 0.0009). In addition, having hypertension was not significant in the univariate model but was significant in the multivariate analysis (*p* = 0.017) (Supplemental Table 5). The significant risk factors for cardiovascular-related mortality in NSTEMI subjects, identified with the multivariate model, were age (*p* = 0.0036), being female (*p* = 0.038), being from SEA (*p* = 0.001), and being from the MEACAT region (*p* = 0.034) (Supplemental Table 6).

## Discussion

Previous studies have revealed age, diabetes, hypertension, smoking, peripheral artery disease, chronic liver disease, chronic renal disease, history of stroke, history of cancer, and chronic obstructive pulmonary disease as being potential risk factors for all-cause mortality in post-MI patients [9–16]. In the current study, we also found older age, low LVEF, female gender, increased time from MI to hospital admission, being from SEA, and the presence of CAD as potential risk factors for all-cause mortality. Ye et al. showed LVEF dysfunction and presence of pump failure to be potential risk factors for all-cause mortality after MI [16]. In another study, Drybus et al. found the risk of all-cause mortality post-MI to be higher in patients with stage 3/4 chronic kidney disease, diabetes, or hypercholesterolemia [17]. Vega et al. also showed that patients with elevated blood pressure and diabetes were at higher risk of all-cause death following MI [18]. Hence, valid prediction models for patients with post-MI mortality is essential and must consider different variables and comorbidities that influence heart disease [19].

Of the deaths in our study, the most common cause of death was SCD, which is not surprising given that SCD remains the most common type of cardiac mortality post-MI [20]. Also, SCD made up more than 75% of all cardiovascular deaths in our cohort and so the risk factors for cardiovascular mortality revealed in our analysis can be used as a surrogate for SCD risk in this population. In regards to risk factors for cardiac mortality, we found increased age, reduced LVEF, and being from SEA to be the strongest predictors of cardiac-related death for 1-year post-MI. Age is a key factor in predicting post-MI mortality, specifically because older individuals are more likely to experience vascular complications after an MI [16, 21, 22]. Reduced LVEF was also an independent risk factor for 1-year cardiac-related death in our analysis. This agrees with previous studies where an LVEF < 40% is a strong predictor of cardiac death post-MI [16, 23]. This finding further supports timely treatment of heart failure patients with low LVEF using β-blockers, angiotensin-converting enzyme inhibitors, and ICD therapy to decrease the mortality rate of patients with MI [24].

Interestingly, we also show that individuals from SEA are at a higher risk for cardiac-related death post-MI. Previous studies have documented a higher rate of cardiac-related deaths in SEA than in other regions [25, 26]. Additionally, cardiac-related deaths occur 5 to 10 years earlier in affected individuals from SEA than those from Western countries [25, 27]. A previous study also found a higher prevalence of CAD, diabetes, hypertension, and ischemic heart failure in SEA countries [28]. This has raised the hypothesis that SEA has a unique propensity for MI that is not accounted for by traditional risk variables [25]. Higher rates of smoking and air pollution have been proposed as contributors to the higher incidences of cardiac disease in SEA, with smoking being identified as a major risk factor for MI [25]. These previous results are in line with our finding that those individuals from SEA who experience an MI are at higher risk for death than post-MI patients from other regions.

Also, we found that female gender is a risk factor for all-cause mortality but not cardiovascular mortality in post-MI patients. This is an interesting finding that implies that females in our cohort experienced higher rates of non-cardiac deaths or “unknown” deaths and is worthy of further investigation. It has been shown that in Asian countries the rate of cardiovascular death is significantly higher in men than women [29]. This explains why female gender was not associated with cardiovascular death, and also helps explain the high rate of males in the trial who experienced an MI compared to females.

Lastly, we found no overlap in risk factors between STEMI and NSTEMI patients for either all-cause or cardiovascular-related death within 1-year post-MI. Contrary to our results, a previous analysis of 2,151 patients from France found similar risk factors between STEMI and NSTEMI patients, notably increased age and diabetes, indicating possible geographical differences [30]. Takeji et al. showed that within 6 months of MI, NSTEMI patient deaths were more often caused by weakened post-resuscitation status or HF, while STEMI patient deaths were more often a result of mechanical cardiac complications or cardiogenic shock [31]. Thus, differences in the death etiology may help explain the contrast in risk factors between STEMI and NSTEMI groups given that most of the deaths in our study occurred within 6 months of MI.

As previously mentioned, adoption of ICD therapy in qualified patients from regions included in this analysis is low. Several factors contribute to this low rate including cost, patient education, and lack of resources to name a few. While the current analysis cannot eliminate these barriers to access, they help inform a clinician’s decision to refer post-MI patients with reduced LVEF for further assessment of risks. The guidelines recommend an ICD for post-MI patients with a reduced LVEF more than 40 days post-MI and, at the very least, these individuals should be referred for SCD risk stratification and management [32]. If these patients also hold any of the risk factors identified in this study, they may be at even increased risk and thus referral is even more imperative.

### Limitations

The biggest limitation of this study was the small sample size. Although the primary Improve SCA Bridge study had a large sample size, the number of patients who died represented a small subset, which limited the strength of our risk factors model given the number of characteristics considered as potential risk factors. This likewise limited our ability to appropriately test the proportionality of hazards to ensure that variables met assumptions for the multivariate Cox proportional-hazards model. The follow-up duration of this study was also short, which limited our ability to capture more deaths and develop a more robust analysis. Also, there were many patients that exited the study if not referred for further risk stratification and management or were lost to follow-up, which also reduced the number of deaths to be analyzed. Other limitations included the retrospective nature of the analysis, high percentage of males, which could limit the generalizability of the results, and number of deaths that were classified as “unknown”. Given that this study was performed with patients from specific regions on the Asian continent, the generalizability of the results onto other populations outside of these regions is limited and not recommended. Future studies utilizing large patient databases or a longer follow-up period to capture more deaths would allow for a more robust analysis of mortality risk factors in post-MI patients from these regions. Also a future analysis looking at the risks specific to each geography or the impact of differing healthcare structures across countries would be meaningful.

## Conclusion

Low LVEF, increased age, and being from SEA were predictors of both all-cause and cardiovascular-related mortality in patients from the Improve SCA Bridge study. Additionally, being female, presence of CAD, and increased MI to hospital time were predictors of cardiovascular-related death post-MI. Post-MI patients in these regions who possess these characteristics are potentially at heightened risk of SCD and, therefore, SCD risk stratification and management strategies should reflect this increased risk to improve outcomes in regions where ICD use is low. While all patients in the original study should have been referred to an electrophysiologist, per guidelines, the results of this sub-analysis identify several subgroups who would have especially benefited from referral.

### Supplementary Information

Additional file 1 (DOCX 43 KB)

## Data Availability

All data generated or analyzed during this study are included in this published article and its supplementary information files.

## Abbreviations

CAD: Coronary artery disease
ICD: Implantable cardioverter defibrillators
LVEF: Left ventricular ejection fraction
MEACAT: Middle East, Africa, Central Asia and Turkey
MI: Myocardial infarction
NSTEMI: Non‐ST-segment elevation myocardial infarction
SCA: Sudden Cardiac Arrest
SCD: Sudden cardiac death
SEA: Southeast Asia
STEMI: ST-elevated myocardial infarction
