# Supplementary material for: Risk factors for mortality in post-myocardial infarction patients: insights from the improve SCA bridge study
Source: Egypt Heart J. 2024 Jun 7;76:72. doi: 10.1186/s43044-024-00505-2 (PMC11161447; doi:10.1186/s43044-024-00505-2)
Supplement: Supplementary file 1 — Additional file 1 (DOCX 43 KB) [file 43044_2024_505_MOESM1_ESM.docx]

**SUPPLEMENTAL INFORMATION:**

**Supplemental Table 1. Exclusion Criteria**

| **Exclusion Criteria** |
| --- |
| - Patient previously received or is currently implanted with an ICD or CRT-D - Patient has a contraindication for a ICD/CRT-D - Patient’s life expectancy is < 12 months - Patient who has had an EP referral within the last 12 months - Any exclusion criteria required by local law (e.g. pregnancy or breast feeding etc.) - Patient is unable (e.g. mental disorder) or unwilling to be compliant with the responsibilities as specified in the informed consent form (see supplemental material of primary study). - Patient is enrolled in a concurrent study that has not been approved for concurrent enrollment by the Medtronic Clinical Trial Leader |

ICD = implantable cardioverter defibrillator, CRT-D = cardiac resynchronization therapy-defibrillator, EP = electrophysiologist

**Supplemental Table 2. Baseline Characteristics, STEMI vs. Non-STEMI**

| **Subject Characteristics** | **STEMI (N = 985)** | **Non-STEMI (N = 503)** | **Overall (N = 1491)** |
| --- | --- | --- | --- |
| **Age (years)** |  |  |  |
| Mean ± SD | 58.7 ± 12.0 | 63.2 ± 11.4 | 60.2 ± 12.0 |
| **Gender (N, %)** |  |  |  |
| Male | 840 (85.3%) | 386 (76.7%) | 1228 (82.4%) |
| **ST elevation** |  |  |  |
| STEMI (N, %) | 985 (100.0%) | 0 (0.0%) | 985 (66.1%) |
| **NYHA classification (N, %)** |  |  |  |
| Subject Does Not Have Heart Failure | 634 (64.4%) | 270 (53.7%) | 906 (60.8%) |
| Class I | 41 (4.2%) | 24 (4.8%) | 65 (4.4%) |
| Class II | 130 (13.2%) | 59 (11.7%) | 189 (12.7%) |
| Class III | 67 (6.8%) | 53 (10.5%) | 121 (8.1%) |
| Class IV | 38 (3.9%) | 42 (8.3%) | 80 (5.4%) |
| NYHA Classification Not Available | 75 (7.6%) | 55 (10.9%) | 130 (8.7%) |
| **LVEF at baseline** |  |  |  |
| Mean ± SD | 39.8 ± 6.6 | 39.7 ± 7.1 | 39.8 ± 6.8 |
| Median | 40.0 | 40.0 | 40.0 |
| Minimum - Maximum | 4 - 50 | 9 - 50 | 4 - 50 |
| **Door to Balloon Time (Hours)** |  |  |  |
| Subjects With Measure Available (N, %) | 723 (73.4%) | 341 (67.8%) | 1066 (71.5%) |
| Mean ± SD | 29.9 ± 108.5 | 41.9 ± 65.8 | 33.8 ± 97.0 |
| Median | 1.3 | 16.8 | 2.6 |
| Minimum - Maximum | -79 - 1386 | -18 - 461 | -79 - 1386 |
| **Time MI to Hospital Admission (Days)** |  |  |  |
| Mean ± SD | 0.9 ± 2.1 | 1.4 ± 2.6 | 1.1 ± 2.3 |
| Median | 0.2 | 0.4 | 0.2 |
| Minimum - Maximum | -1 - 15 | -9 - 17 | -9 - 17 |
| **Diabetes** | 323 (32.8%) | 240 (47.7%) | 564 (37.8%) |
| Type I | 26 (2.6%) | 8 (1.6%) | 34 (2.3%) |
| Type II | 297 (30.2%) | 233 (46.3%) | 531 (35.6%) |
| **Cancer** | 20 (2.0%) | 16 (3.2%) | 36 (2.4%) |
| **COPD** | 17 (1.7%) | 16 (3.2%) | 33 (2.2%) |
| **Renal Dysfunction** | 44 (4.5%) | 69 (13.7%) | 113 (7.6%) |
| **CHF** | 60 (6.1%) | 34 (6.8%) | 94 (6.3%) |
| **CAD** | 346 (35.1%) | 190 (37.8%) | 539 (36.2%) |
| **Hypertension** | 431 (43.8%) | 309 (61.4%) | 742 (49.8%) |
| **PVD** | 14 (1.4%) | 20 (4.0%) | 35 (2.3%) |
| **Prior Stroke** | 39 (4.0%) | 26 (5.2%) | 65 (4.4%) |
| CAD = coronary artery disease; CHF = congestive heart failure; COPD = Chronic obstructive pulmonary disease; ISC = India Subcontinent; LVEF = left ventricular ejection fraction; MEACAT = Middle East, Africa, Central Asia and Turkey; PVD = peripheral vascular disease; SD = Standard deviation; SEA = Southeast Asia; STEMI = ST elevated myocardial infarction | | | |

**Supplemental Table 3.** **Risk Factors for All-Cause Mortality in STEMI Patients**

|  | | | **Univariate Model** | | **Multivariate Model*** | |
| --- | --- | --- | --- | --- | --- | --- |
| **Characteristics** | **N**  **(Counts)** | **Mean Value**  **(N=984)** | **Hazard Ratio**  **(95% CI)** | **P-Value** | **Hazard Ratio**  **(95% CI)** | **P-Value** |
| **Age (years)** |  | 58.7 | 1.032  (1.007, 1.058) | 0.0124 | 1.020  (0.994, 1.047) | 0.1369 |
| **LVEF (%)** |  | 39.8 | 0.914  (0.886, 0.942) | <.0001 | 0.909  (0.878, 0.942) | <.0001 |
| **Door to Balloon Time (Hours)** |  | 29.9 | 1.000  (0.996, 1.004) | 0.9257 |  |  |
| **Time MI to Hospital Admission (Min)** |  | 1339.2 | 1.000  (1.000, 1.000) | 0.2996 |  |  |
| **Female** | 145 |  | 1.894  (0.960, 3.738) | 0.0656 | 1.949  (0.942, 4.033) | 0.0720 |
| **China** | 257 |  | 2.375  (1.312, 4.299) | 0.0043 | (Reference for Geographic Regions) | |
| **Korea** | 147 |  | 0.559  (0.200, 1.561) | 0.2670 |  | |
| **SEA** | 145 |  | 1.645  (0.815, 3.322) | 0.1650 | 2.089  (0.976, 4.471) | 0.0579 |
| **ISC** | 236 |  | 0.624  (0.290, 1.341) | 0.2269 |  |  |
| **MEACAT** | 132 |  | 0.587  (0.210, 1.639) | 0.3092 |  |  |
| **Taiwan** | 68 |  | No Patients Died | 0.9824 |  |  |
| **Diabetes** | 323 |  | 1.029  (0.553, 1.912) | 0.9288 |  |  |
| **Cancer** | 20 |  | No Patients Died | 0.9853 |  |  |
| **COPD** | 17 |  | 2.646  (0.641, 10.924) | 0.1786 |  |  |
| **Renal** | 44 |  | 2.867  (1.131, 7.268) | 0.0265 | 2.047  (0.791, 5.297) | 0.1396 |
| **CHF** | 60 |  | 0.657  (0.159, 2.715) | 0.5622 |  |  |
| **CAD** | 346 |  | 2.782  (1.533, 5.052) | 0.0008 | 2.726  (1.464, 5.076) | 0.0016 |
| **Hypertension** | 431 |  | 1.977  (1.089, 3.589) | 0.0252 | 1.579  (0.848, 2.943) | 0.1499 |
| **PVD** | 14 |  | 1.643  (0.226, 11.925) | 0.6235 |  |  |
| **Prior Stroke** | 39 |  | 2.476  (0.887, 6.912) | 0.0835 | 2.510  (0.866, 7.276) | 0.0902 |

CAD = coronary artery disease; CHF = congestive heart failure; CI = confidence interval; COPD = Chronic obstructive pulmonary disease; ISC = India Subcontinent; LVEF = left ventricular ejection fraction; MEACAT = Middle East, Africa, Central Asia and Turkey; PVD = peripheral vascular disease; SEA = Southeast Asia; STEMI = ST elevated myocardial infarction

**Supplemental Table 4.** **Risk Factors for Cardiovascular Mortality in STEMI Patients**

|  | | | **Univariate Model** | | **Multivariate Model*** | |
| --- | --- | --- | --- | --- | --- | --- |
| **Characteristics** | **N**  **(Counts)** | **Mean Value**  **(N=984)** | **Hazard Ratio**  **(95% CI)** | **P-Value** | **Hazard Ratio**  **(95% CI)** | **P-Value** |
| **Age (years)** |  | 58.7 | 1.019  (0.988, 1.051) | 0.2252 |  |  |
| **LVEF (%)** |  | 39.8 | 0.907  (0.874, 0.942) | <.0001 | 0.911  (0.873, 0.949) | <0.0001 |
| **Door to Balloon Time (Hours)** |  | 29.9 | 1.000  (0.996, 1.004) | 0.9771 |  |  |
| **Time MI to Hospital Admission (Min)** |  | 1339.2 | 1.000  (1.000, 1.000) | 0.2186 |  |  |
| **Female** | 145 |  | 0.937  (0.326, 2.692) | 0.9033 |  |  |
| **China** | 257 |  | 1.438  (0.654, 3.164) | 0.3664 | (Reference for Geographic Regions) | |
| **Korea** | 147 |  | 0.914  (0.318, 2.627) | 0.8678 |  | |
| **SEA** | 145 |  | 1.835  (0.784, 4.296) | 0.1619 | 2.124  (0.853, 5.290) | 0.1058 |
| **ISC** | 236 |  | 0.929  (0.396, 2.177) | 0.8651 |  |  |
| **MEACAT** | 132 |  | 0.449  (0.107, 1.888) | 0.2744 |  |  |
| **Taiwan** | 68 |  | No Patients Died | 0.9859 |  |  |
| **Diabetes** | 323 |  | 1.082  (0.503, 2.326) | 0.841 |  |  |
| **Cancer** | 20 |  | No Patients Died | 0.9882 |  |  |
| **COPD** | 17 |  | 2.033  (0.277, 14.934) | 0.4856 |  |  |
| **Renal** | 44 |  | 3.618  (1.258, 10.405) | 0.017 | 3.133  (1.071, 9.168) | 0.0371 |
| **CHF** | 60 |  | 1.058  (0.252, 4.453) | 0.9383 |  |  |
| **CAD** | 346 |  | 2.626  (1.254, 5.499) | 0.0104 | 2.869  (1.320, 6.234) | 0.0078 |
| **Hypertension** | 431 |  | 1.408  (0.680, 2.917) | 0.3571 |  |  |
| **PVD** | 14 |  | 2.565  (0.349, 18.858) | 0.3546 |  |  |
| **Prior Stroke** | 39 |  | 0.904  (0.123, 6.647) | 0.9213 |  |  |

Abbreviations same as in Supplemental Table 1

**Supplemental Table 5. Risk Factors for All-Cause Mortality in NSTEMI Patients**

|  | | | **Univariate Model** | | **Multivariate Model*** | |
| --- | --- | --- | --- | --- | --- | --- |
| **Characteristics** | **N**  **(Counts)** | **Mean Value**  **(N=503)** | **Hazard Ratio**  **(95% CI)** | **P-Value** | **Hazard Ratio**  **(95% CI)** | **P-Value** |
| **Age (years)** |  | 63.2 | 1.054  (1.027, 1.083) | <0.0001 | 1.059  (1.031, 1.088) | <0.0001 |
| **LVEF (%)** |  | 39.7 | 1.010  (0.966, 1.055) | 0.6680 |  |  |
| **Door to Balloon Time (Hours)** |  | 41.9 | 1.003  (0.996, 1.010) | 0.3912 |  |  |
| **Time MI to Hospital Admission (Min)** |  | 2038.2 | 1.000  (1.000, 1.000) | 0.0007 | 1.000  (1.000, 1.000) | 0.0026 |
| **Female** | 137 |  | 2.562  (1.383, 4.749) | 0.0028 | 1.865  (0.989, 3.516) | 0.0541 |
| **China** | 90 |  | 2.857  (1.560, 5.233) | 0.0007 | (Reference for Geographic Regions) | |
| **Korea** | 51 |  | 0.378  (0.117, 1.223) | 0.1044 |  |  |
| **SEA** | 110 |  | 2.841  (1.396, 5.782) | 0.0040 | 4.061  (1.943, 8.490) | 0.0002 |
| **ISC** | 65 |  | 0.247  (0.076, 0.798) | 0.0195 |  |  |
| **MEACAT** | 50 |  | 0.794  (0.312, 2.022) | 0.6284 |  |  |
| **Taiwan** | 240 |  | No Patients Died |  |  |  |
| **Diabetes** | 16 |  | 2.504  (1.302, 4.817) | 0.0060 | 3.364  (1.642, 6.891) | 0.0009 |
| **Cancer** | 16 |  | 0.746  (0.103, 5.422) | 0.7724 |  |  |
| **COPD** | 69 |  | 0.768  (0.106, 5.580) | 0.7939 |  |  |
| **Renal** | 34 |  | 2.007  (0.987, 4.082) | 0.0545 |  |  |
| **CHF** | 190 |  | No Patients Died |  |  |  |
| **CAD** | 309 |  | 1.278  (0.696, 2.347) | 0.4284 |  |  |
| **Hypertension** | 20 |  | 0.777  (0.423, 1.428) | 0.4168 | 0.456  (0.239, 0.869) | 0.0170 |
| **PVD** | 26 |  | 0.584  (0.080, 4.248) | 0.5954 |  |  |
| **Prior Stroke** | 117 |  | 1.412  (0.436, 4.571) | 0.5646 |  |  |

Abbreviations same as in Supplemental Table 1

**Supplemental Table 6. Risk Factors for Cardiovascular Mortality in NSTEMI Patients**

|  | | | **Univariate Model** | | **Multivariate Model*** | |
| --- | --- | --- | --- | --- | --- | --- |
| **Characteristics** | **N**  **(Counts)** | **Mean Value**  **(N=503)** | **Hazard Ratio**  **(95% CI)** | **P-Value** | **Hazard Ratio**  **(95% CI)** | **P-Value** |
| **Age (years)** |  | 63.2 | 1.054  (1.015, 1.094) | 0.0062 | 1.060  (1.019, 1.103) | 0.0036 |
| **LVEF (%)** |  | 39.7 | 0.971  (0.919, 1.025) | 0.2857 |  |  |
| **Door to Balloon Time (Hours)** |  | 41.9 | 1.002  (0.991, 1.013) | 0.7054 |  |  |
| **Time MI to Hospital Admission (Min)** |  | 2038.2 | 1.000  (1.000, 1.000) | 0.4931 |  |  |
| **Female** | 137 |  | 3.276  (1.391, 7.718) | 0.0066 | 2.568  (1.055, 6.250) | 0.0377 |
| **China** | 90 |  | 0.868  (0.318, 2.370) | 0.7826 | (Reference for Geographic Regions) | |
| **Korea** | 51 |  | 0.503  (0.117, 2.158) | 0.3548 |  |  |
| **SEA** | 110 |  | 3.552  (1.378, 9.157) | 0.0087 | 5.529  (1.996, 15.316) | 0.001 |
| **ISC** | 65 |  | 0.560  (0.165, 1.901) | 0.3522 |  |  |
| **MEACAT** | 50 |  | 1.971  (0.721, 5.389) | 0.1859 | 3.247  (1.090, 9.672) | 0.0344 |
| **Taiwan** | 240 |  | No Patients Died | 0.9909 |  |  |
| **Diabetes** | 16 |  | 2.239  (0.904, 5.548) | 0.0816 | 2.418  (0.936, 6.243) | 0.0681 |
| **Cancer** | 16 |  | No Patients Died | 0.9916 |  |  |
| **COPD** | 69 |  | 1.535  (0.206, 11.443) | 0.6757 |  |  |
| **Renal** | 34 |  | 1.514  (0.509, 4.499) | 0.4557 |  |  |
| **CHF** | 190 |  | No Patients Died | 0.9879 |  |  |
| **CAD** | 309 |  | 0.783  (0.316, 1.940) | 0.5970 |  |  |
| **Hypertension** | 20 |  | 0.853  (0.359, 2.024) | 0.7177 | 0.499  (0.202, 1.231) | 0.1314 |
| **PVD** | 26 |  | 1.207  (0.162, 8.990) | 0.8545 |  |  |
| **Prior Stroke** | 117 |  | 0.891  (0.120, 6.635) | 0.9099 |  |  |

Abbreviations same as in Supplemental Table 1
